# Reduced order analytical modelling of micro wind turbine rotordynamics with tower shadow effects

**DOI:** 10.1038/s41598-025-00364-0

**Published:** 2025-05-13

**Authors:** Aly A. Ramzy, Adel Elsabbagh, Ashraf M. Hamed, Ahmed A. Barakat

**Affiliations:** 1 Design and Production Engineering Department, Faculty of Engineering, Ain Shams University, Cairo, Egypt; 2 Mechanical Power Engineering Department, Faculty of Engineering, Ain Shams University, Cairo, Egypt; 3 School of Computation, Information and Technology, Technical University of Munich, Munich, Germany

**Keywords:** Micro wind turbines, Vibration analysis, Gyroscopic effects, Tower shadow effects, Design parametric study, Mechanical engineering, Wind energy

## Abstract

Micro scale wind turbines (μWTs) in the order of 10 kW or less, suffer high vibration levels compared to larger ones. This can be attributed to the fact that they rotate at higher rotational speeds. For simplicity, blades are directly attached to a permanent magnet generator (PMG) of an outer rotor type with no need to install a gearbox. Also due to their small size, a tail vane represents a cost-effective solution for aligning the turbine with the wind direction. Those tail vanes generate significant yaw rates as a consequence of the unpredictable variations in wind direction. High yaw rates, along with increased rotational speeds, generate considerable gyroscopic loads. Therefore, μWTs involve a unique dynamic loading condition. Considering that the existing software packages are primarily designed for large wind turbines, a thorough investigation into the dynamics of μWTs is essential. In this study, we address the peculiarity of dynamics of μWTs using a simple mathematical model that helps in providing better dynamical insights than numerical calculations. Using the mathematical model, a parametric study is conducted involving two parameters: the generator’s bearing span and the position of the rotor’s centre of gravity (CG) along the rotor axis. The parametric study revealed that increasing the bearing span yields a decrease in vibrations. Also, shifting the centre of gravity of the rotor towards the generator’s rear bearing reduces vibrations across all considered degrees of freedom, except for the rotor’s vertical vibrations and pitching, which achieve a minimum value. An optimal placement of the centre of gravity is calculated and used afterwards to improve the design of an existing μWT. A case study highlights this improvement in terms of root mean square vibrations. The presented model can thereby help designers to build μWTs with better performance and longer lifespan offering a better understanding of the dynamical effects of design parameters

## Introduction

Wind energy is gaining more interest in scientific research community due to its potential to replace other energy sources such as fossil fuels^1^. This will help to reduce the rate of increase in the Earth’s surface temperature^2^ and will restrict severe ramifications^3^. On the large scale, wind farms continue to be installed providing huge amounts of power owing to the large areas swept by the rotors of the large wind turbines^4^. However, in many rural areas which do not have high density of population, utility scale wind turbines are not economical in comparison to micro and small wind turbines, micro wind turbines are defined as those having an output power of 10 kW or less^5^. Since μWTs are not necessarily placed at high-wind-speed locations, they are designed to work at low Reynold’s numbers^6^. Hence, obtaining comparable tip speed ratios to those of large wind turbines requires μWTs to operate at high rotational speeds. This contrast between small and large wind turbines is manifested in a multitude of design and dynamical aspects^7^. In terms of the yaw mechanism, in both types it is crucial to maintain maximal efficiency by aligning the rotor with respect to the wind direction. Two types of yaw mechanisms are being implemented: active yaw for real-time alignment; and passive yaw, which normally depends on the design of the tail vane. The former is used for large wind turbines since the rotor mass is quite large and hence will require high yawing torque to overcome the high inertia loads, while the latter is conveniently used in μWTs^6^. Another distinction is also found between small and large wind turbines regarding rotational speeds. Given low rotational speeds of large turbines, a gear box is deployed to magnify the rotational speed before reaching the generator^8^. However, the relatively high rotational speeds in μWTs allow for fastening the blades directly to a permanent magnet generator of outer rotor type^6^.

The previously discussed design considerations in μWTs imply a different dynamical case in comparison with large turbines. The usage of passive yaw system in μWTs together with the stochastic nature of the wind yields very high yaw rates^9^. Moreover, both high rotational speeds and high yaw rates result in high gyroscopic loads. Gyroscopic moments act in a plane that is perpendicular to the precession motion plane. This means when yawing occurs, gyroscopic moments cause pitching of the rotor up and down^10^. The stress on the main shaft is found to be dominated by gyroscopic moment as specified by^5,6^. Due to the aforementioned design and dynamical differences between small and large wind turbines, applying the results of the research carried out on large wind turbines on small ones cannot be sufficient without proper investigation.

Wind turbine blades, either for small or large wind turbines, have been heavily studied in the past years^11–17^. Recently, structural health monitoring techniques were applied to monitor the vibrations of small wind turbine blades. Experiments revealed that variations in ambient temperature have a significant impact on the vibration characteristics of the blades^18^. Additionally, a numerical extension to the experimental work was conducted to address a wider range of load scenarios that are difficult to replicate in the laboratory^19^. Gyroscopic moments were studied in few papers on large, but not small, wind turbines considering the whole structure and its dynamics^20,21^. Stol et al. studied the modal behaviour of a two-bladed teetered-rotor turbine using Floquet analysis^20^. They stated that gyroscopic coupling drives the system into instability in the absence of damping and stiffness in the yaw and teeter directions. Velazquez and Swartz presented a study on a wind turbine using rotating finite elements^21^. They stated that for low rotating speeds, gyroscopic moment is very low compared to the moment caused by aerodynamic forces. However, when the rotor speed increases, gyroscopic effects vary the natural frequencies of the system, thus decreasing the critical speeds.

Few studies were made concerning the dynamics and vibrations of the μWT structure. Castellani et al. studied the tower vibrations of a μWT numerically using FAST (Fatigue, Aerodynamics, Structures, Turbulence code developed at the National Renewable Energy Laboratory NREL) and validated it experimentally^22^. They found that the vibration of the tower is significantly affected by the electromechanical, tower shadow and unbalance excitations. Castellani et al. showed that there exists an overestimation of the thrust coefficient from Blade Element Momentum (BEM) and FAST simulation tool due to neglecting the effect of blade deformation on tower shadow^23^. Their experimental results show that small blade deflections cause a considerable increase in the tower shadow effect. Vedovelli et al. conducted a numerical and experimental study on the effect of blade numbers on dynamic response of μWT and showed that the 5-bladed turbine produces the same performance curve at lower rotational speeds than that of the 3-bladed turbine hence, lower noise and vibrations^24^.

Furthermore, the use of outer rotor generators in μWTs unfolds a different dynamical behaviour in comparison to large turbines. On one hand, they simplify the construction of the μWT by allowing the direct installation of the rotor blades to the casing of the generator. On the other hand, outer rotor generators are characterized by their small width compared to the diameter of the generator and the rotor in general. This results in excessive vibrations of the rotor relative to the axis of the shaft. In turn, those excessive vibrations lead to several problems such as deteriorating the performance of the turbine, producing less power, causing fatigue of the structural parts, and hence a shorter life of the whole μWT.

As a first attempt to limit severe consequences, several signal processing techniques were conducted for condition monitoring of μWT generators focusing on the mechanical faults such as bearings^25,26^. Additionally, recent studies have been conducted to evaluate the limits, with a focus on the early detection of bearing faults^27^. The most probable cause of these faults is the high reactions on the bearings and the vibrations of the rotor. Accordingly, since on one hand, the dynamical studies performed on large wind turbines could not be extended to small ones due to the aforementioned peculiarities, while on the other hand, μWT dynamics are not properly addressed in most design software since they are more tailored to utility scale turbines, separate studies for the dynamics of μWTs are still required. Thus, this distinction between large and small turbines with respect to dynamics in addition to the lack of sufficient studies for the latter creates the main motivation for this study.

This study addresses, therefore, the question of the dynamical modelling specified for μWTs for the sake of discussing their distinctive features in a class of systems that should be separated from large WTs due to the issues mentioned above. To this end, we developed a mathematical model that predicts the vibrational behaviour of μWTs at different operating conditions, considering the centrifugal forces and the tower shadow effect. Although not all degrees of freedom, and their accompanying dynamical influences, are yet included, the present study is verified through comparisons with reported experimental and numerical data in the literature with a respective discussion. Furthermore, relying on the fidelity of the developed model, it can be extended to other non-reported cases. Moreover, a parametric study is conducted on the proposed model, in addition to a numerical case study to demonstrate the efficacy of using the model as a tool for designing μWTs.

The paper is organized as follows: Section “Methodology” describes the methodology of how the lumped parameter model was obtained. Section “Time varying forces” focuses on the time-varying forces acting on the turbine and the methods used to determine them. Section “Equations of motion” provides the mathematical model of the problem. Section “Model verification” focuses on verifying the model through a comparison with experimental data presented in^22^. In Section “Parametric study”, a two-variable parametric study is conducted. Section “Case study” presents the application of the model on a case study. Finally, Section “Conclusion” provides the conclusions.

## Methodology

**Fig. 1:**
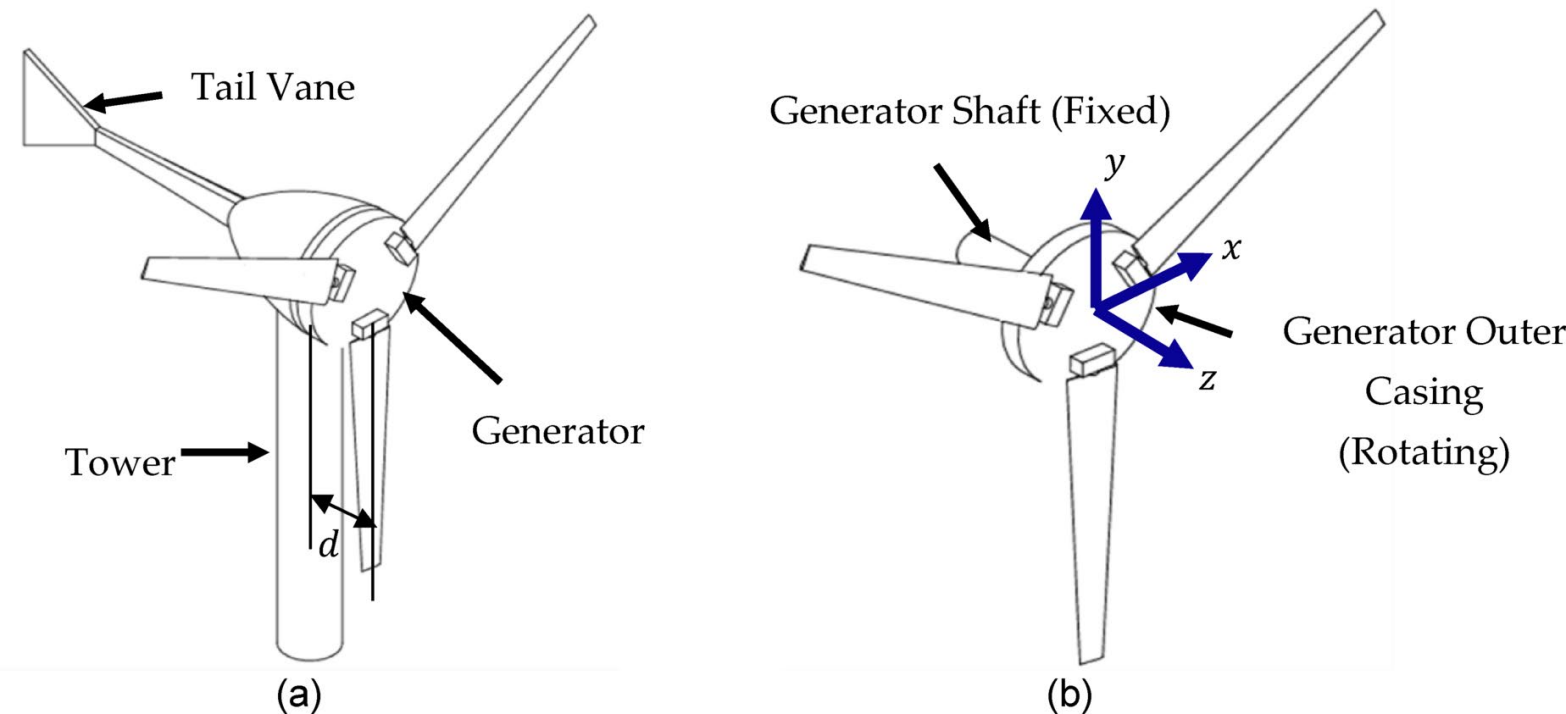
A three-dimensional model of the μWT showing the rotor, tower and tail in (**a**) and the non-inertial reference frame fixed to the rotor in (**b**).

**Fig. 2:**
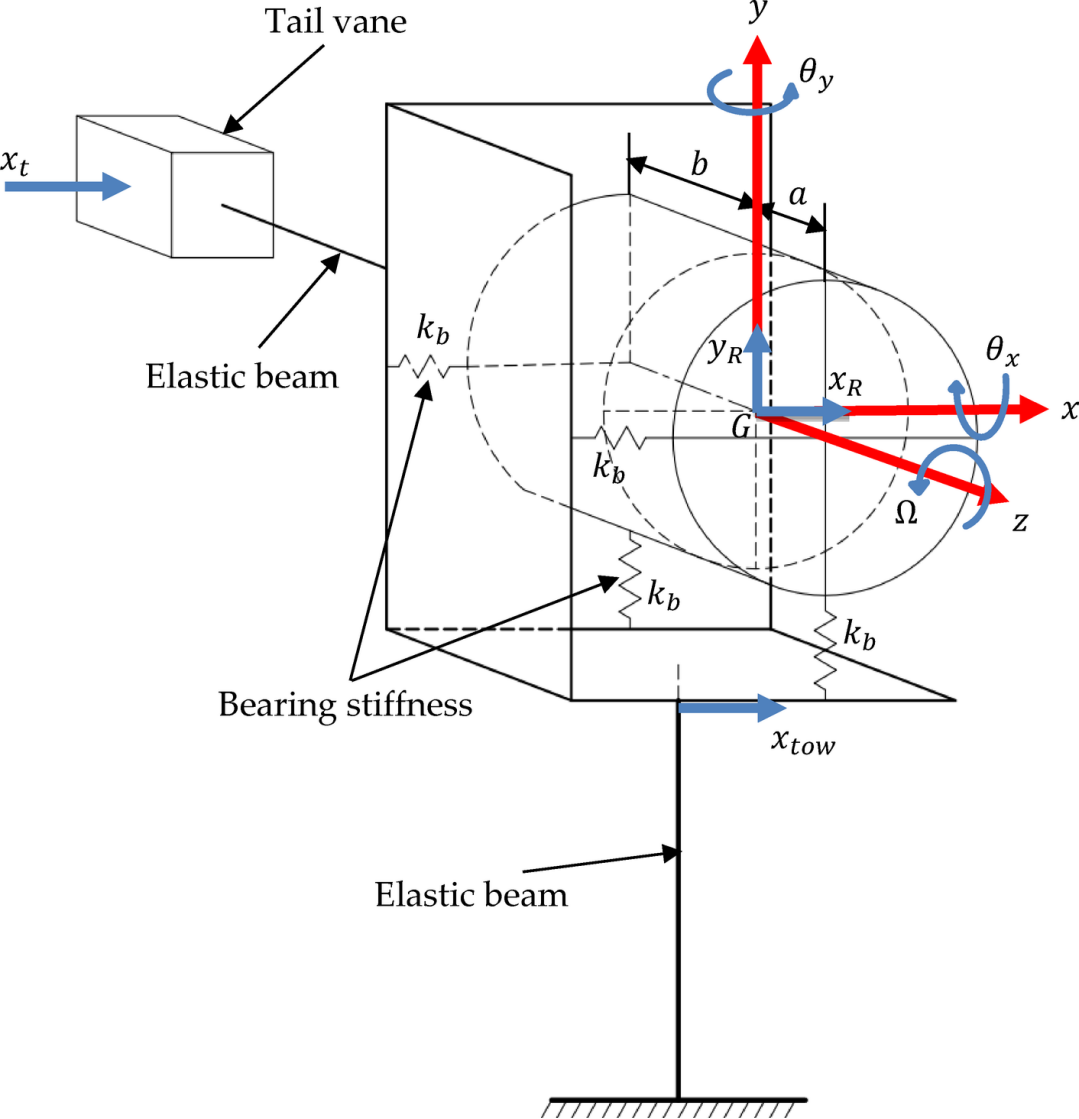
A lumped parameter model of the μWT, showing the respective degrees of freedom considered.

In this work, a micro wind turbine with a permanent magnet generator and a passive yawing mechanism is considered. Rotor blades are fixed to the generator’s rotating outer casing forming the rotor, as shown in Fig. 1b while the generator shaft (the stator) remains fixed. For the sake of developing a mathematical model for the vibrations of μWTs, a lumped parameter system is given. The bearings of the generator are modelled as axisymmetric elastic springs with two orthogonal components of stiffness ${k}_{b}$, the rotor’s mass ${m}_{R}$ is formed by the generator together with the blades and the braking disc, and the mass moments of inertia about the $x$,$y$ and $z$ axes are given the values ${I}_{G},{I}_{G}$ and $J$, respectively. Figure 2 shows a schematic for the simplified lumped parameter model representing the main components of the μWT.

The tower and tail are considered as elastic beams. The tower’s vibrational degrees of freedom include the bending rotation around the $x$ and $z$ axes, as well as the displacement along the $y$ axis. The same applies for the tail, where its rotation is allowed around the $x$ and $y$ axes with a buckling-free displacement in the $z$ direction. Furthermore, due to their relatively high frequencies, the compression modes are excluded. Moreover, the flow of vibrational energy, from the rotor, limits the possible vibration modes to those in the $x$ direction. This relies on the fact that the construction of the rotor is almost rigid in the $z$ direction, thus, the coupling of the tower bending around the $x$ axis to the rotor in this direction would not yield any interesting dynamics.

The same applies for tail bending. This leaves us with only translational degrees of freedom in the $x$ direction (${x}_{t}$ and ${x}_{tow}$) resulting from the bending of the tail and tower around the $y$ and $z$ axes, respectively. As shown by Fig. 2, this model accounts for six degrees of freedom: two degrees of freedom for the rotor’s translation ${x}_{R},{y}_{R}$; two for its rotation ${\theta }_{x},{\theta }_{y}$; in addition to the displacements of the tower ${x}_{tow}$ and that of the tail vane ${x}_{t}.$ The mass of the tail vane is denoted by ${m}_{t}$, while ${k}_{b}$ denotes the bearing stiffness, ${m}_{R}$ denotes the rotor mass, ${k}_{t}$ denotes the tail stiffness and $\Omega$ is the rotor rotational speed, which is assumed to be constant. Considering the tower vibrations with its first cantilever bending mode, its effective mass is calculated according to

1 $$m_{tow} = 0.24 \rho_{tow} V_{tow}$$

where ${\rho }_{tow}$ and ${V}_{tow}$ are the tower’s density and volume, respectively^28^.

## Time varying forces

Several dynamic forces act on μWTs. In this study, only two main forces are considered; (1) centrifugal forces due to rotor unbalance, and (2) variations in the aerodynamic forces due to tower shadow.

Arising from misalignment, centrifugal forces are calculated as ${F}_{c}={m}_{R}e{\Omega }^{2}$, where ${m}_{R}$ is the total rotor mass, $e$ the eccentricity, which is the shift between the rotor’s centre of mass and centre of geometry, and $\Omega$ the rotation speed.

Tower shadow effects, on the other hand, arises due to the deceleration of the wind in front of the tower causing the blade to experience minimum wind speed whenever it passes in the upwind region. Accordingly, the aerodynamic forces acting on the rotor varies with a frequency that is equal to the number of blades multiplied by the rotational speed of the rotor. To calculate the time varying aerodynamic forces due to tower shadow, one can use the blade element momentum (BEM) technique together with the formula deduced for the variation in wind speed due to tower shadow in^29^, which is given by

2 $$\nu_{tow} \left( {r,\psi ,d} \right) = V_{0} r_{tow}^{2} \frac{{r^{2} \sin^{2} \left( \psi \right) - d^{2} }}{{\left( {r^{2} \sin^{2} \left( \psi \right) + d^{2} } \right)^{2} }} ,$$

where ${V}_{0}$ is the spatial mean wind speed, $\psi$ the azimuth angle, ${r}_{tow}$ the tower radius, $r$ the radial distance on the blade measured from the rotor centre, and $d$ the gap between the blades and the tower (see Fig. 1a). For simplification we can assume that the spatial mean wind speed is equal to the hub height wind speed $V_{H}$^30^. The lift $dF_{L}$ and drag $dF_{D}$ forces acting on a blade element of width $dr$ are given by

3 $$dF_{D} \left( r \right) = \frac{1}{2} C_{D} \rho_{air} c V_{rel}^{2} dr,$$

4 $$dF_{L} \left( r \right) = \frac{1}{2} C_{L} \rho_{air} c V_{rel}^{2} dr,$$

where ${C}_{D}$ and ${C}_{L}$ are the drag and lift coefficients respectively, $c$ is the chord length and ${V}_{rel}=\sqrt{{V}_{wind}^{2}+{\left(\Omega r\right)}^{2}}$ is the wind speed relative to the blade.

**Fig. 3:**
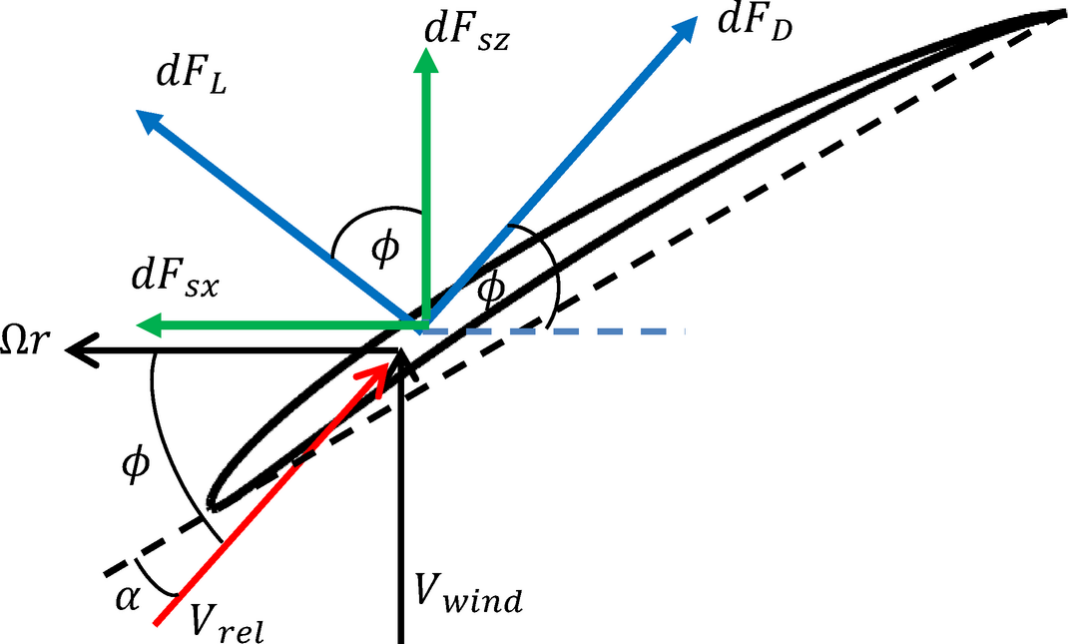
Airfoil geometry with aerodynamic forces.

To account for the tower shadow disturbance, we substitute for ${V}_{wind}$ by

5 $$V_{wind} = V_{H} + \nu_{tow} \left( {r,\psi ,d} \right) = V_{H} \left( {1 + r_{tow}^{2} \frac{{r^{2} \sin^{2} \left( \psi \right) - d^{2} }}{{\left( {r^{2} \sin^{2} \left( \psi \right) + d^{2} } \right)^{2} }}} \right),$$

which allows for calculating the time varying aerodynamic forces in the $x$ and $z$ directions (see Fig. 3) according to

6 $$\tan \left( \phi \right) = \frac{{V_{wind} }}{{{\Omega }r}},$$

7 $$dF_{sx} \left( r \right) = dF_{L} \left( r \right)\sin \left( \phi \right) - dF_{D} \left( r \right)\cos \left( \phi \right),$$

8 $$dF_{sz} \left( r \right) = dF_{L} \left( r \right)\cos \left( \phi \right) + dF_{D} \left( r \right)\sin \left( \phi \right).$$

By summing over the $n$ blade elements

9 $$F_{sx} = \mathop \sum \limits_{i = 1}^{n} dF_{sx} ,\;\;F_{sz} = \mathop \sum \limits_{i = 1}^{n} dF_{sz} ,$$

where $\phi$ is the angle between $\Omega r$ and ${V}_{rel}$. Table 1 summarizes the used aerodynamic properties.

**Table 1:** The parameters used for calculating the time varying aerodynamic forces.

Rotor diameter	1.6 m
Airfoil type	E63
Designed angle of attack,${\alpha }_{design}$	5 deg
Hub height wind speed,${V}_{H}$	6.5 m/s
Tower radius,${r}_{tow}$	7.5 cm
Air density,${\rho }_{air}$	1.225 kg/m^3^
Number of elements,$n$	781

**Fig. 4:**
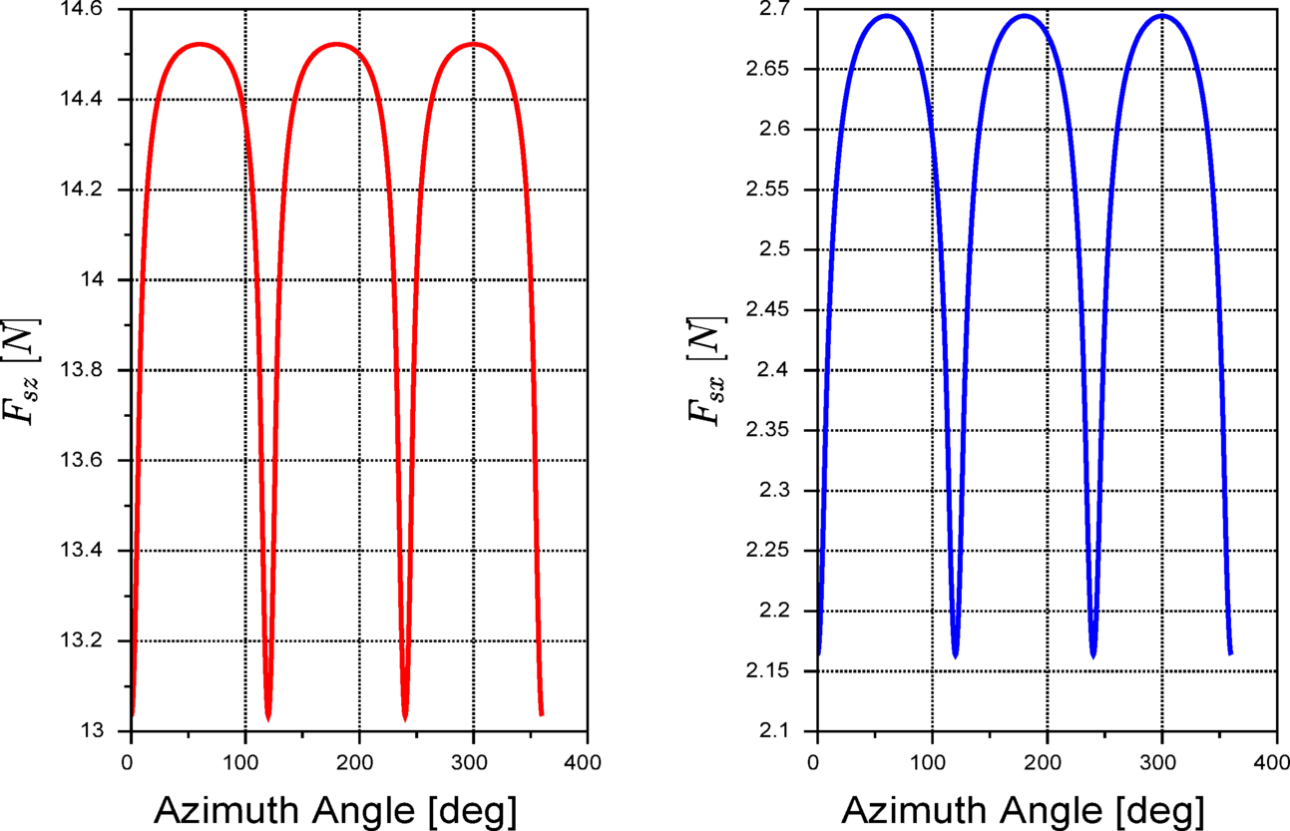
The azimuthal variation of aerodynamic forces according to Eq. (9) using the parameters in Table 1.

Figure 4 shows a plot of Eq. (9) over one rotor revolution. As shown in the figure, the aerodynamic forces drop down three times, corresponding to the decrease in the wind velocity with respect to each blade.

## Equations of motion

The system of differential equations governing the vibrations of the above-mentioned model is obtained using Lagrange’s method^31^. To obtain the kinetic energy of the rotor, the sequence of Euler angles is chosen to be ${\theta }_{x}, {\theta }_{y}, \psi =\Omega t$.

Let $\hat{U}={\left\{\hat{\mathbf{I}},\hat{\mathbf{J}},\hat{\mathbf{K}}\right\}}^{T}$ be the global axes unit vector triad in the $x$, $y$, and $z$ directions while $\hat{u}={\left\{\hat{\mathbf{i}},\hat{\mathbf{j}},\hat{\mathbf{k}}\right\}}^{T}$ be a non-inertial unit vector triad attached to the rotor. By following the above-mentioned sequence of transformations, we get the following:

10 $$\hat{u}^{\prime } = R_{x} \hat{U}$$

11 $$\hat{u}^{\prime \prime } = R_{y} \hat{u}^{\prime }$$

12 $$\hat{u} = R_{z} \hat{u}^{\prime \prime }$$

13 $$\hat{u} = R_{z} R_{y} R_{x} \hat{U}$$

where ${R}_{x}$, ${R}_{y}$ and ${R}_{z}$ are rotation matrices about $x$, $y$ and $z$ axes, respectively. The rotor angular velocity vector can be written as:

14 $$\vec{\omega } = \dot{\theta }_{x} {\hat{\mathbf{I}}} + \dot{\theta }_{y} {\hat{\mathbf{j}}}^{\prime } + {\Omega } {\hat{\mathbf{k}}} = \omega_{x} {\hat{\mathbf{i}}} + \omega_{y} {\hat{\mathbf{j}}} + \omega_{z} {\hat{\mathbf{k}}} ,$$

where the rotor’s angular velocity about the non-inertial triad $\hat{u}$ is found to be:

15 $$\begin{gathered} \omega_{x} = \dot{\theta }_{x} \cos \theta_{y} {\text{cos}}\psi + \dot{\theta }_{y} {\text{sin}}\psi \hfill \\ \omega_{y} = - \dot{\theta }_{x} \cos \theta_{y} \sin \psi + \dot{\theta }_{y} \cos \psi \hfill \\ \omega_{z} = \dot{\theta }_{x} \sin \theta_{y} + {\Omega } \hfill \\ \end{gathered}$$

Accordingly, one can calculate the rotor’s kinetic energy as

16 $$T_{rotor} = \frac{1}{2}m_{R} \left( {\dot{x}_{R}^{2} + \dot{y}_{R}^{2} } \right) + \frac{1}{2}I_{G} \left( {\dot{\theta }_{x}^{2} + \dot{\theta }_{y}^{2} } \right) + \frac{1}{2}J\left( {\Omega^{2} + 2\dot{\theta }_{x} \theta_{y} \Omega } \right),$$

using small angle approximation.

The kinetic energies for the tower and tail vibrations are then given by

17 $$T_{tower} = \frac{1}{2}m_{tow} \dot{x}_{tow}^{2} ,\;\;T_{t} = \frac{1}{2}m_{t} \dot{x}_{t}^{2}$$

By summing the kinetic energies and considering the potential energy of the system as the sum of the strain energies in all the springs, the Lagrangian reads as

18 $$\begin{gathered} {\mathcal{L}} = \frac{1}{2}m_{tow} \dot{x}_{tow}^{2} + \frac{1}{2}m_{t} \dot{x}_{t}^{2} + \frac{1}{2}m_{R} \left( {\dot{x}_{R}^{2} + \dot{y}_{R}^{2} } \right) + \frac{1}{2}I_{G} \left( {\dot{\theta }_{x}^{2} + \dot{\theta }_{y}^{2} } \right) \hfill \\ \quad \quad + \frac{1}{2}J\left( {\Omega^{2} + 2\dot{\theta }_{x} \theta_{y} \Omega } \right) - \frac{1}{2}k_{tow} x_{tow}^{2} - \frac{1}{2}k_{t} \left( {x_{t} - x_{tow} } \right)^{2} \hfill \\ \quad \quad - \frac{1}{2}k_{b} \left( {a\theta_{y} + x_{R} - x_{tow} } \right)^{2} - \frac{1}{2}k_{b} \left( {x_{R} - b\theta_{y} - x_{tow} } \right)^{2} \hfill \\ \quad \quad - \frac{1}{2}k_{b} \left( {y_{R} - a\theta_{x} } \right)^{2} - \frac{1}{2}k_{b} \left( {y_{R} + b\theta_{x} } \right)^{2} \hfill \\ \end{gathered}$$

Using Lagrange’s equations of motion

$$\frac{d}{dt}\left( {\frac{{\partial {\mathcal{L}}}}{{\partial \dot{q}_{i} }}} \right) - \frac{{\partial {\mathcal{L}}}}{{\partial q_{i} }} = Q_{i} ,\;\;q_{i} \in \left[ {x_{tow} , x_{R} , y_{R} , \theta_{x} , \theta_{y} , x_{t} } \right]$$

**Fig. 5:**
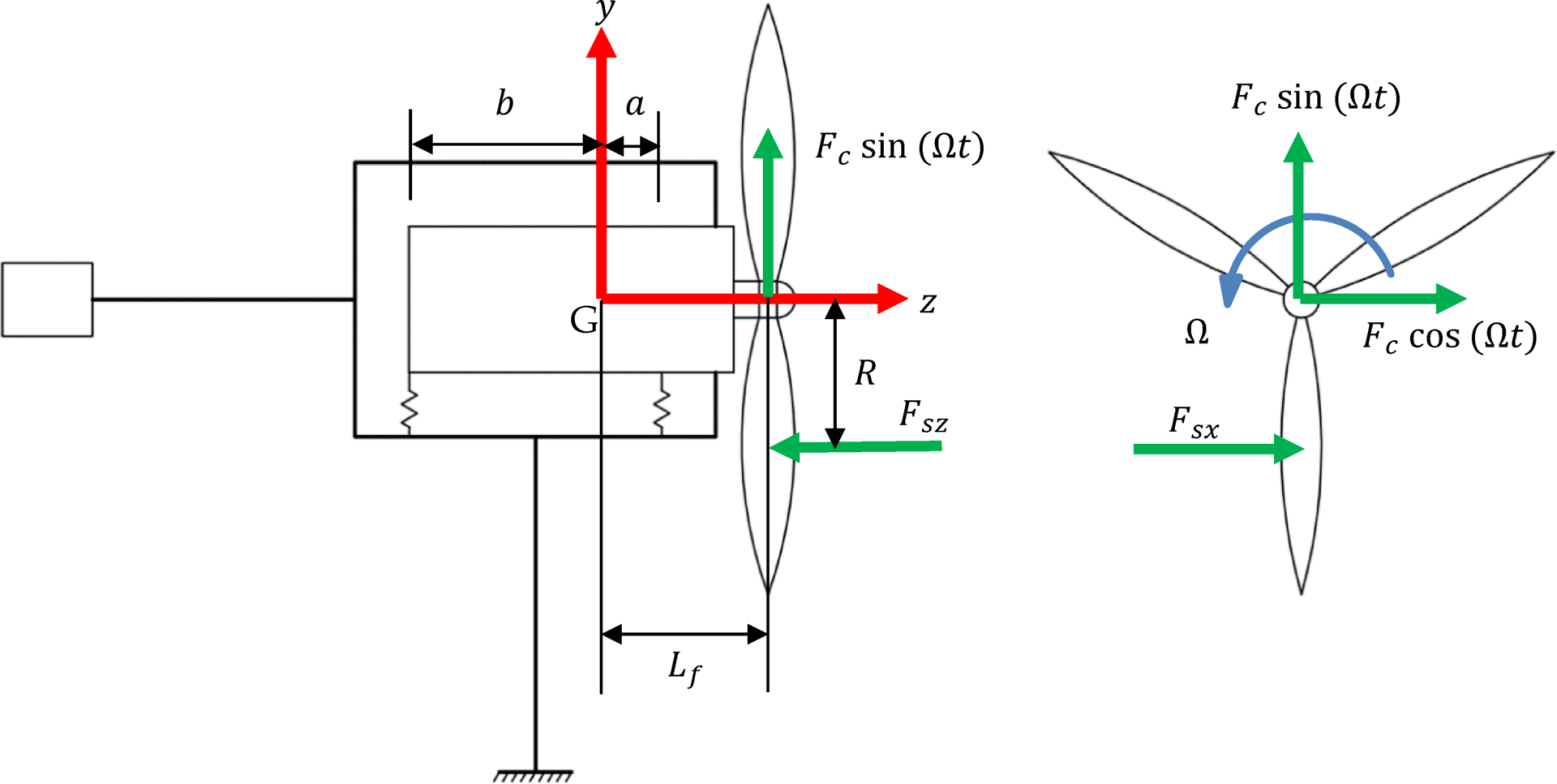
Schematic figure showing the time varying forces acting on the wind turbine.

In Fig. 5, one should note that the rotor mass ${m}_{R}$ is concentrated at the centre of gravity of the rotor located at distances $a$ and $b$ away from the front and rear bearings, respectively. Hence, for convenience, the following non-dimensional parameters and relations are introduced:from which, one gets the following system of differential equations

$$L_{r} = a + b,\;\;\left( \right)^{*} = \frac{\left( \right)}{{L_{r} }},\;\;\overline{\left( \right)} = \frac{\left( \right)}{{L_{r}^{2} }},$$

19 $$m_{tow} \ddot{x}^{*}_{tow} + \left( {k_{tow} + 2k_{b} + k_{t} } \right)x_{tow}^{*} - 2k_{b} x_{R}^{*} + k_{b} \left( {b^{*} - a^{*} } \right)\theta_{y} - k_{t} x_{t}^{*} = 0,$$

20 $$m_{R} \ddot{x}^{*}_{R} + 2k_{b} x_{R}^{*} - 2k_{b} x_{tow}^{*} - k_{b} \left( {b^{*} - a^{*} } \right)\theta_{y} = F_{2} ,$$

21 $$m_{R} \ddot{y}^{*}_{R} + 2k_{b} y_{R}^{*} + k_{b} \left( {b^{*} - a^{*} } \right)\theta_{x} = F_{3} ,$$

22 $$\overline{{I_{G} }} \ddot{\theta }_{x} + \bar{J}\Omega \dot{\theta }_{y} + k_{b} \left( {a^{{*2}} + b^{{*2}} } \right)\theta _{x} + k_{b} \left( {b^{*} - a^{*} } \right)y_{R}^{*} = F_{4} ,$$

23 $$\overline{{I_{G} }} \ddot{\theta }_{y} - \bar{J}\Omega \dot{\theta }_{x} + k_{b} \left( {a^{{*2}} + b^{{*2}} } \right)\theta _{y} + k_{b} \left( {b^{*} - a^{*} } \right)x_{{tow}}^{*} - k_{b} \left( {b^{*} - a^{*} } \right)x_{R}^{*} = F_{5} ,$$

24 $$m_{t} \ddot{x}^{*}_{t} + k_{t} x_{t}^{*} - k_{t} x_{tow}^{*} = 0.$$

In matrix form, this yields a six degree-of-freedom linear system of differential equations

25 $${\mathbf{M}} {\mathbf{\ddot{u}}} + \left( {{\mathbf{C}} + {\mathbf{G}}} \right){\dot{\mathbf{u}}} + {\mathbf{K}} {\mathbf{u}} = {\mathbf{F}}\left( {\mathbf{t}} \right),$$

where $\mathbf{M}$, $\mathbf{G}$ and $\mathbf{K}$ are mass, gyroscopic, and stiffness matrices, respectively and $\mathbf{C}$ is Rayleigh’s proportional damping matrix with ${a}_{0}$ and ${a}_{1}$ being mass-proportional and stiffness-proportional damping coefficients, respectively, and are taken as ${a}_{0}=0$ and ${a}_{1}=0.0008$. The matrices are given as following:

$${\mathbf{u}} = \left\{ {x_{tow}^{*} , x_{R}^{*} , y_{R}^{*} , \theta_{x} , \theta_{y} , x_{t}^{*} } \right\}^{T}$$

26 $$\begin{gathered} {\mathbf{M}} = \left[ {\begin{array}{*{20}c} {m_{{tow}} } & 0 & 0 & 0 & 0 & 0 \\ 0 & {m_{R} } & 0 & 0 & 0 & 0 \\ 0 & 0 & {m_{R} } & 0 & 0 & 0 \\ 0 & 0 & 0 & {\overline{{I_{G} }} } & 0 & 0 \\ 0 & 0 & 0 & 0 & {\overline{{I_{G} }} } & 0 \\ 0 & 0 & 0 & 0 & 0 & {m_{t} } \\ \end{array} } \right]\;\;{\mathbf{G}} = \left[ {\begin{array}{*{20}c} 0 & 0 & 0 & 0 & 0 & 0 \\ 0 & 0 & 0 & 0 & 0 & 0 \\ 0 & 0 & 0 & 0 & 0 & 0 \\ 0 & 0 & 0 & 0 & {\bar{J}\Omega } & 0 \\ 0 & 0 & 0 & { - \bar{J}\Omega } & 0 & 0 \\ 0 & 0 & 0 & 0 & 0 & 0 \\ \end{array} } \right] \hfill \\ {\mathbf{K}} = \left[ {\begin{array}{*{20}c} {k_{{tow}} + 2k_{b} + k_{t} } & { - 2k_{b} } & 0 & 0 & {k_{b} \left( {b^{*} - a^{*} } \right)} & { - k_{t} } \\ { - 2k_{b} } & {2k_{b} } & 0 & 0 & { - k_{b} \left( {b^{*} - a^{*} } \right)} & 0 \\ 0 & 0 & {2k_{b} } & {k_{b} \left( {b^{*} - a^{*} } \right)} & 0 & 0 \\ 0 & 0 & {k_{b} \left( {b^{*} - a^{*} } \right)} & {k_{b} \left( {a^{{*2}} + b^{{*2}} } \right)} & 0 & 0 \\ {k_{b} \left( {b^{*} - a^{*} } \right)} & { - k_{b} \left( {b^{*} - a^{*} } \right)} & 0 & 0 & {k_{b} \left( {a^{{*2}} + b^{{*2}} } \right)} & 0 \\ { - k_{t} } & 0 & 0 & 0 & 0 & {k_{t} } \\ \end{array} } \right]. \hfill \\ {\mathbf{C}} = a_{0} {\mathbf{M}} + a_{1} {\mathbf{K}} \hfill \\ \end{gathered}$$

With the aid of Fig. 5$\mathbf{F}\left(\mathbf{t}\right)$ is giveny

27 $${\mathbf{F}}\left( {\mathbf{t}} \right) = \left[ {\begin{array}{*{20}c} 0 \\ {\frac{1}{{L_{r} }}\left[ {F_{sx} + F_{c} \cos \left( {{\Omega }t} \right)} \right]} \\ {\frac{1}{{L_{r} }}[F_{c} {\text{sin}}\left( {{\Omega }t} \right)]} \\ {\frac{1}{{L_{r}^{2} }}\left[ { - F_{c} \sin \left( {\Omega t} \right) L_{f} + F_{sz} R} \right]} \\ {\frac{1}{{L_{r}^{2} }}[F_{c} \cos \left( {\Omega t} \right) L_{f} + F_{sx} L_{f} ]} \\ 0 \\ \end{array} } \right]$$

## Model verification

**Fig. 6:**
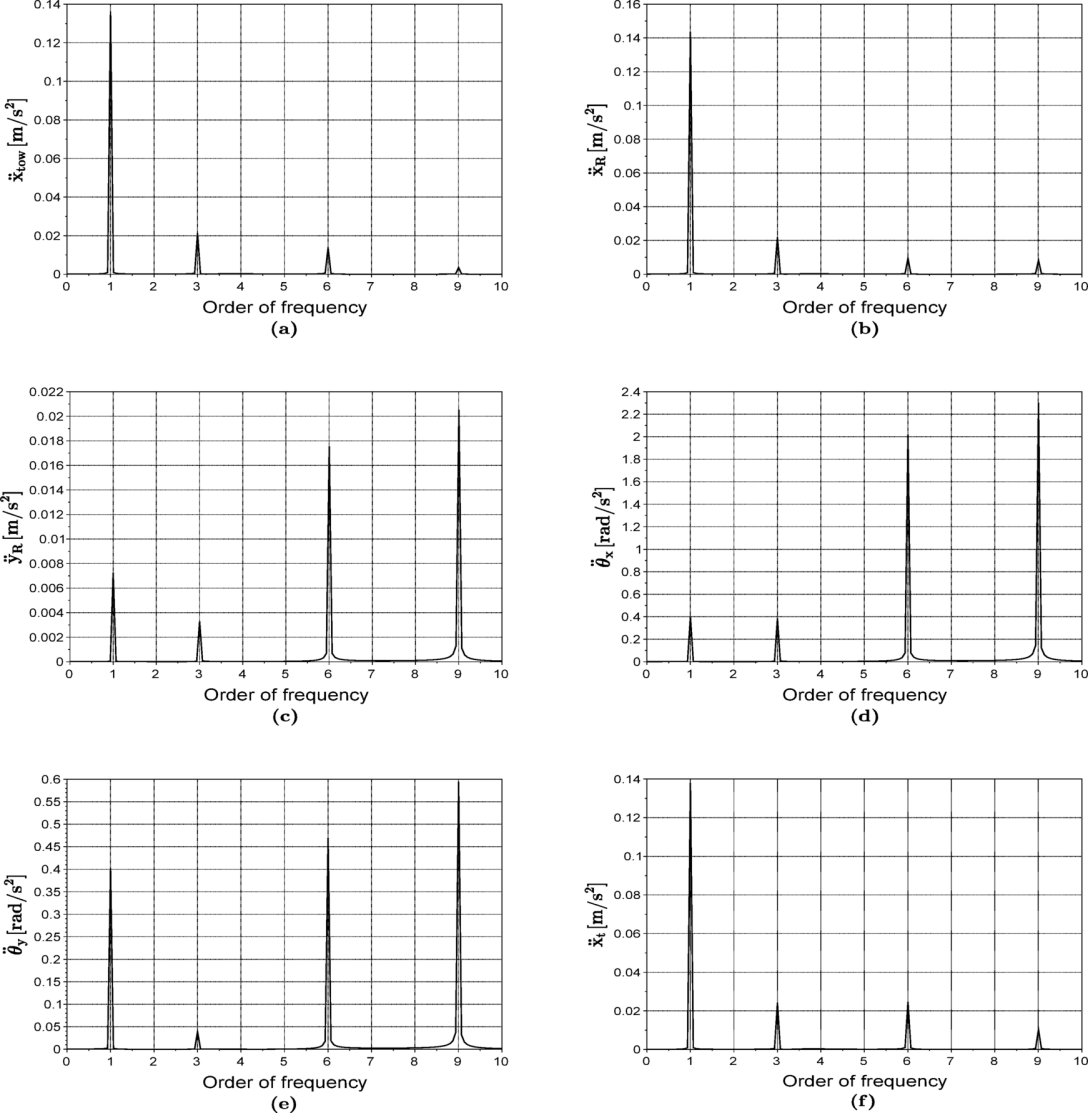
Frequency spectra of the vibrations of the rotor along the x (**b**), y (**c**), pitch ${\theta}_{x}$ (**d**) and yaw ${\theta}_{y}$ (**e**) directions, in addition to those of the tower (**a**) and the tail (**f**) along the x direction.

For the parameters listed in Table 2, the eigenfrequencies of the proposed model are calculated as 184.3, 347.3, 384.4, 429, 1928, and 2325 rad/s. The system of differential equations is solved using ode function implemented in SCILAB. Figure 6 shows the amplitude of vibrations versus the order of frequency, which is equal to the ratio of the Fourier frequency component with respect to the rotational speed. For verification purposes, the predictions of the present model for the tower vibrations are compared to those of Castellani^22^. As seen from Fig. 6, there exist frequencies of order $1p$ (one time per cycle) which corresponds to rotor unbalance, since it’s varying at an angular frequency equal to the rotor’s rotational speed. Additionally, frequencies of order $3p$ and its multiples correspond to the tower shadow forces, as explained before, where the factor of three is related to the number of blades. However, the multiples of $3p$ occur since variation of the aerodynamic loads due to tower shadow is not purely harmonic, thus having Fourier expansion with frequency orders of $3p$, $6p$, $9p$, etc.

**Table 2:** Parameters of the μWT used in the model verification and the parametric study.

Parameter	${m}_{R}$ kg	${k}_{b}$ MN/m	$e$ mm	$\Omega$ rad/s	$a$ mm	$b$ mm	${k}_{tow}$ MN/m	${k}_{t}$ kN/m	${m}_{tow}$ kg	${m}_{t}$ kg	${I}_{G}$ kg m^2^	$J$ kg m^2^	${L}_{f}$ mm
Value	4	7.416	3.2	48.75	23	40	0.45	45	8.883	0.25	0.11	0.084	95

The vibration amplitude corresponding to each of these harmonics is dependent on the magnitude of the excitation, the different compliances of the system’s degrees of freedom and the nearness of the excitation frequency to any of the system’s eigenfrequencies. In general, the tower shadow effects, contributing with the $3p$ harmonics and their multiples, are far less in magnitude than the eccentric mass excitation exhibited in the $1p$ component. This is indeed reflected in the vibrations occurring in the $x$ direction, namely, ${x}_{tow}, {x}_{t}$ and ${x}_{R}$ as shown in Fig. 6a, b and f. In this direction, the low-stiffened tower vibrations ${x}_{tow}$ are transferred to the rotor ${x}_{R}$ and the tail ${x}_{t}$ without much amplification since the bearings between the tower and the rotor have far higher stiffnesses compared to the tower’s at its tip. This applies as well to the tail’s dynamic stiffness at the off-resonant $1p$ frequency. In contrast, the stiffnesses of all the turbine’s elements are relatively high in the $y$ direction, this makes way for the minor tower shadow effects to prevail as can be seen in ${y}_{R}$ vibrations in Fig. 6c. The $9p$ and $6p$ components are highly manifested than the $3p$ in the ${\theta }_{x},{\theta }_{y}$ and ${y}_{R}$ degrees of freedom as shown in Fig. 6c–e. This relates to the fact that three of the system’s eigenfrequencies (347.3, 384.4 and 429 rad/s in this example) lie between the excitation frequencies of $6p$ and $9p$ for the operating speed 48.75 rad/s.

**Fig. 7:**
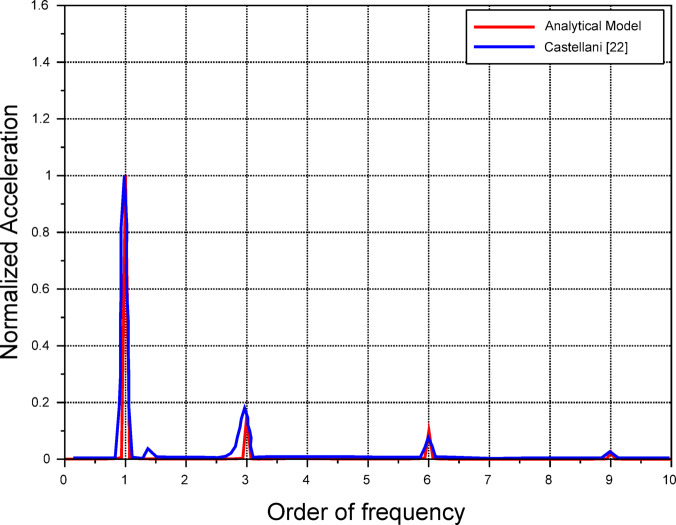
Comparison between the proposed model and the results from Castellani^22^ concerning tower vibration degree of freedom ${x}_{tow}.$

The verification process involved tuning the eccentricity parameter $e$ to match the amplitude of the $1p$ component of our results with the FAST calculations in^22^. Note here that the FAST analysis is validated against experimental data in^22^. Both spectra are then normalised with respect to the $1p$ component and presented in Fig. 7. Both spectra are found to have a good agreement at an eccentricity value of $e = 3.2$ mm.

One can notice that the proposed model is preserving the physics of the experiment conducted in^22^ as the ratios between amplitudes are following the same trend. The deviations may be attributed to the fact that maximum amplitudes are sensitive to the values of damping factors which were not provided by the experiment. Moreover, the blade deflection is not accounted for in the present model while in reality, the gap between the tower and the blade narrows, the impact of the tower shadow effect becomes significantly pronounced. Also, the difference in aerodynamic characteristics between both turbines may contribute to those deviations.

According to the presented comparisons between the predictions of the proposed model with the experimental results in^22^, one can see that the proposed model is verified with respect to the proportionality of the amplitudes at each respective frequency order. This allows us, therefore, to predict μWT vibrations with reasonable accuracy.

## Parametric study

**Fig. 8:**
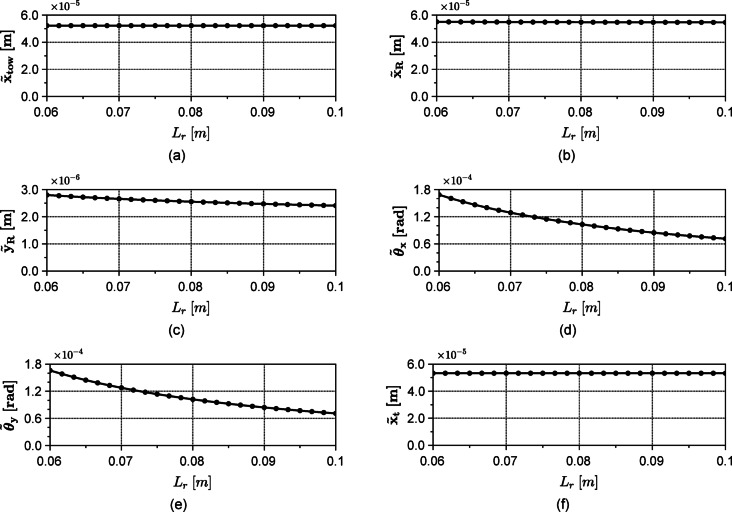
Effect of varying the span length on the vibrations of the rotor along the x (**b**), y (**c**), pitch ${\theta}_{x}$ (**d**) and yaw ${\theta}_{y}$ (**e**) directions, in addition to those of the tower (**a**) and the tail (**f**) along the x direction.

For the sake of understanding the role of the model design parameters in μWT design, a parametric study is conducted by varying, the span between the bearings ${L}_{r}$, and ${a}^{*}=a/{L}_{r}$ while fixing other parameters listed in Table 2. The effect of varying the rotor’s rotational speed, $\Omega$, is to either increase or decrease the vibration amplitudes, depending on whether the rotor’s angular velocity is approaching the system’s critical speeds^31^. This dependency is well discussed elsewhere (see^32^) and will not be considered here as a controllable parameter. The rotor’s rotational speed $\Omega$ is set, therefore, to the rated speed of example turbine. Figure 8 shows the effect of varying the span ${L}_{r}$ on the root mean square value (denoted by tilde) of the six degrees of freedom under consideration. As seen from the figure, as the span ${L}_{r}$ increases, the root mean square (RMS) value for the vibrations in each of Fig. 8c, d and e decreases. An increase in the bearing span enhances the rotor’s rotational stiffness about the $x$ and $y$ axes, thereby reducing vibrations. In the $yz$ plane (see Fig. 5), the aerodynamic thrust force ${F}_{sz}$ generates a concentrated moment of magnitude ${F}_{sz }R$ about the $x$ axis. This moment induces bearing reaction forces proportional to ${F}_{sz} R/{L}_{R}$ which diminish as the bearing span ${L}_{r}$ increases resulting in decrease of ${\widetilde{y}}_{R}, {\widetilde{\theta }}_{x}, {\widetilde{\theta }}_{y}$ with ${\widetilde{\theta }}_{y}$ decreasing due to the increase in the rotational stiffness about the $y$ axis.Conversely, in the $xz$ plane, no such moment is present. Instead, the force transmitted to the tower corresponds to the resultant of the bearing reactions, which equals the applied aerodynamic forces ${F}_{sx}$ and ${F}_{c}\text{cos}(\Omega t)$ plus the rotor inertia forces. Consequently, while a larger bearing span reduces individual bearing reactions, it does not significantly influence vibrations along the $x$ direction. One can notice that ${\widetilde{x}}_{tow}$, ${\widetilde{x}}_{R}$ and ${\widetilde{x}}_{t}$ are not significantly affected.

**Fig. 9:**
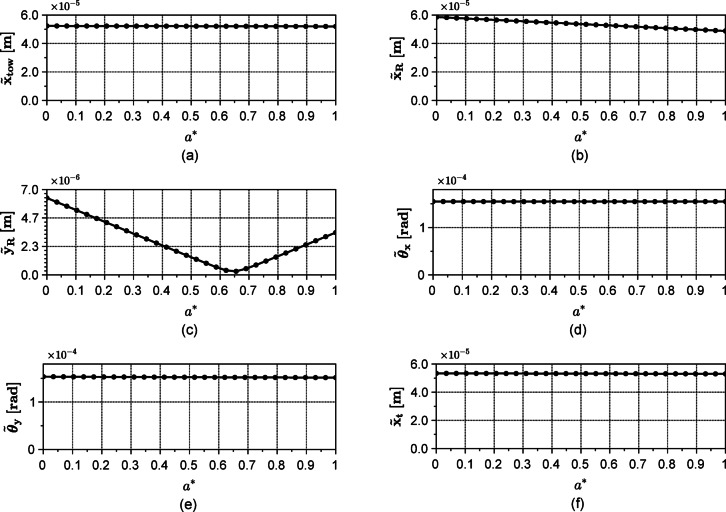
Effect of changing the centre of gravity position on the vibrations of the rotor along the x (**b**), y (**c**), pitch ${\theta}_{x}$ (**d**) and yaw ${\theta}_{y}$ (**e**) directions, in addition to those of the tower (**a**) and the tail (**f**) along the x direction.

Figure 9 shows the effect of varying the parameter ${a}^{*}$ which corresponds to shifting the position of the rotor’s centre of gravity. As ${a}^{*}$ increases, the vibration’s RMS value in all degrees of freedom is slightly affected except for the rotor’s translational degrees of freedom, ${\widetilde{x}}_{R}$ and ${\widetilde{y}}_{R}$,with ${\widetilde{y}}_{R}$ exhibiting a minimal value (Fig. 9c). Concerning the rotor’s vertical vibration ${\widetilde{y}}_{R}$, corresponding to the pitching degree of freedom, has a negative centrifugal term (which is the dominant term in practice) that varies with ${L}_{f}$ and a tower shadow term that does not change by varying ${a}^{*}$ as indicated by the fourth row of Eq. (27). However, the third degree of freedom, corresponding to the rotor’s vertical vibrations, has a positive centrifugal term that does not change with ${a}^{*}$ as shown by the third row of Eq. (27). Accordingly, the rotor’s pitching ${\theta }_{x}$ and vertical vibration ${y}_{R}$ are out of phase with each other. One can see that in the interval $0<{a}^{*}\le 0.5$ the net force ${F}_{3}-{k}_{b}({b}^{*}-{a}^{*}){\theta }_{x}$ in Eq. (21) is decreasing as ${a}^{*}$ increases yielding the decrease in ${\widetilde{y}}_{R}$. However, in the interval $0.5<{a}^{*}\le 1$ the force $-{k}_{b}({b}^{*}-{a}^{*}){\theta }_{x}$ is out of phase with ${F}_{3}$ and its magnitude increases with ${a}^{*}$. This means there is a value for ${a}^{*}$ where the net force ${F}_{3}-{k}_{b}({b}^{*}-{a}^{*}){\theta }_{x}$ magnitude is minimum. According to Fig. 9a, this occurs at ${a}^{*}=0.655$ and hence a minimum ${\widetilde{y}}_{R}$. But as ${a}^{*}$ increases above $0.655$, the previously mentioned net force starts to increase in an opposite phase causing ${\widetilde{y}}_{R}$ to increase once more while being in phase with the rotor pitching ${\theta }_{x}$.

## Case study

The case study in hand is regarding a 1 kW μWT that has been designed in faculty of engineering Ain Shams University with the parameters in Table 2. This turbine participates in the International Small Wind Turbine Contest in Netherlands (ISWTC)^33^. In order to improve the performance of the turbine, we analyse in this work two proposed modifications regarding the PMG for the sake of vibration reduction: $L_{r} ,a^{*} = \left\{ {100\;{\text{mm}},0.655} \right\}, \left\{ {100\;{\text{mm}},1} \right\}$, however, the experimental implementation is kept for a future study The span length is chosen to be significantly large as a result of the parametric study, however, within the practical limit. Hence, these two configurations reflect either the minimization of ${\widetilde{y}}_{R}$ or ${\widetilde{x}}_{R}$ within the domain of ${a}^{*}\in \left[\text{0,1}\right].$

**Fig. 10:**
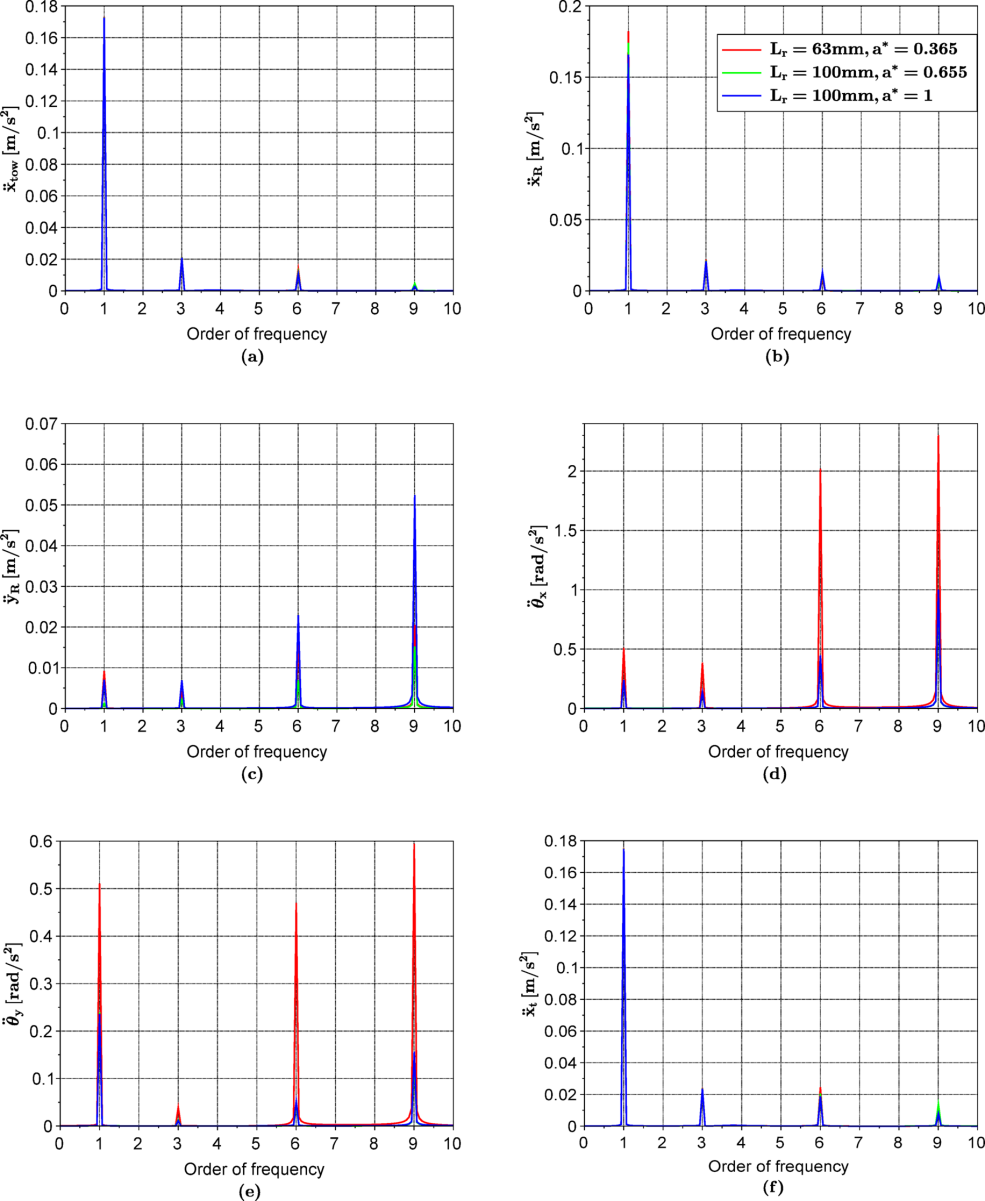
Frequency spectra for the vibrations of the rotor along the x (**b**), y (**c**), pitch ${\theta}_{x}$ (**d**) and yaw ${\theta}_{y}$ (**e**) directions, in addition to those of the tower (**a**) and the tail (**f**) along the x direction, at $\Omega = 48.75$ rad/s for different values of ${L}_{r}$ and ${a}^{*}$.

**Fig. 11:**
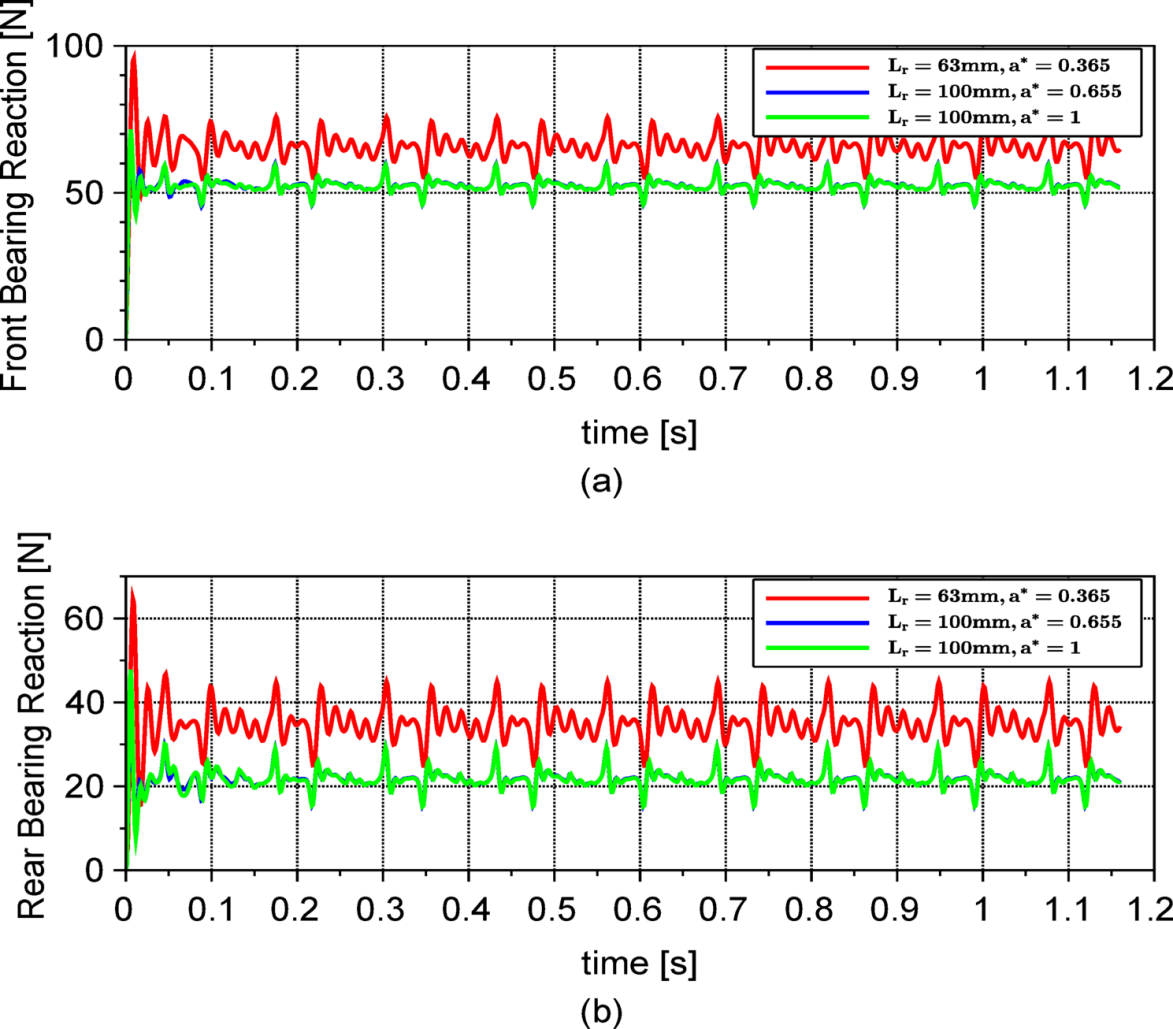
(**a**) Dynamic forces transmitted to the front and (**b**) rear bearings as a function of time.

By solving the system of differential equations for both configurations with respect to the original turbine design parameters, one gets the frequency spectra shown in Fig. 10. It can be shown that the acceleration amplitudes of the rotor’s angular perturbations (${\theta }_{x}$,${\theta }_{y})$ decrease by more than 50% after applying the above-mentioned modifications. However, for the rotor’s vertical vibrations ${y}_{R}$ (Fig. 10c), the amplitudes decreased in the first modification but increased in the second one to be even more than the unmodified case. Meanwhile the vibrations pointing in the $x$ direction (Fig. 10a, b and f) are slightly affected. Accordingly, the parameters to be used for this μWT should be those in the first configuration as it has the smallest ${\ddot{y}}_{R}$. Using the proposed model, we could simply calculate the optimum parameters that minimize the vibrations. However, this is not the only output we can get. Additionally, we can calculate the bearing dynamic reactions as shown in Fig. 11. One can notice how the forces transmitted to the bearings have decreased with the chosen modifications. This can be extremely beneficial to improve the performance of the PMG and increase the lifespan. It is worth mentioning that small values of ${a}^{*}$ are conventional in many μWTs. This is attributed to the fact that the mass of the rotor blades pushes the centre of gravity towards the front bearings. Overcoming this unbalanced distribution of the mass by bringing the centre of gravity close to the middle of the bearing span would have a positive impact on the overall lifespan of the bearings and the μWT in general.

## Conclusion

Micro wind turbines have distinctive dynamical features in comparison to large wind turbines as outlined in the introduction. One example is the outer rotor PMG that are commonly used in μWTs. This is due to their simple design and high rotation speeds that eliminate the need for a gear box between the rotor and the generator. However, one of the disadvantages of these generators from a mechanical point of view is the small span between their roller bearings, which leads to increasing the reaction forces on the bearings. The rotor’s vibrations are hence enlarged, and the lifespan of the generator is reduced. In this study, a simple mathematical model is proposed that is capable of capturing the dynamic behaviour of these turbines and thus paving the way for future studies that particularly address their dynamics using the explanatory power of analytical models in comparison with numerical ones. The model predictions are verified against experimental results in the literature for the case of a μWT exposed to constant wind load and tower shadow effects. A parametric study shows that increasing the bearings span leads to a significant decrease in the angular perturbations of the rotor. Moreover, the location of the rotor’s centre of gravity with respect to the bearings plays an important role in decreasing the rotor’s translational degrees of freedom to a local minimal. The study shows that it is recommended for the rotor’s centre of gravity to be located at approximately 50–60% of the bearings span. This is a tricky task taking into consideration the unsymmetric design of the rotor along the turbine main axis. Applying the findings of the parametric study to an existing μWT, we could calculate the RMS vibrations after modification. This offered an enhancement of more than 50% in the RMS vibrations for some degrees of freedom. Furthermore, the proposed model was able to predict the dynamic forces transmitted to the bearings, thus providing the designers with analytic tool to determine the bearing life with considerably higher accuracy. Generally speaking, given the limited number of the model’s degrees of freedom, this minimal model opens a new avenue in studying other types of forcing or dynamic interactions of relative importance in the design of micro wind turbines with minimal computational expenses and complexity. Such insights have been elusive via numerical methods that are used previously in dynamical studies concerning μWTs.

## Data Availability

All the data used in this manuscript has been presented within the article.

## List of symbols

$a$: Distance between the front bearing and the rotor’s centre of gravity
${a}_{0}$: Mass-proportional damping coefficient
${a}_{1}$: Stiffness-proportional damping coefficient
${\alpha }_{design}$: Designed angle of attack
$b$: Distance between the rear bearing and the rotor’s centre of gravity
$$c$$: Chord length
${C}_{D}$: Coefficient of drag
${C}_{L}$: Coefficient of lift
$\mathbf{C}$: Rayleigh’s damping matrix
$${F}_{c}$$: Centrifugal force
$${F}_{D}$$: Drag force
$${F}_{L}$$: Lift force
${F}_{sx}$: Time-varying tangential force due to tower shadow
${F}_{sz}$: Time-varying thrust force due to tower shadow
$\phi$: The angle between the blade tangential velocity and the relative velocity of the wind
$$\mathbf{G}$$: Gyroscopic matrix
${I}_{G}$: Rotor’s moment of inertia about the $x$ and $y$ axes
$J$: Rotor’s polar moment of inertia
$${k}_{b}$$: Bearing’s stiffness
${k}_{tow}$: Tower effective stiffness
${k}_{t}$: Tail effective stiffness
$$\mathbf{K}$$: Stiffness matrix
${L}_{f}$: Distance between the rotor’s centre of gravity and the blades
${L}_{r}$: Rotor’s bearing span
$$\mathcal{L}$$: System Lagrangian
${m}_{tow}$: Tower effective mass
$${m}_{R}$$: Rotor mass
${m}_{t}$: Tail vane mass
$n$: Number of elements used in blade element momentum
$\Omega$: Rotor’s rotational speed
$${q}_{i}$$: Generalized coordinates
$${Q}_{i}$$: Generalized forces
${\theta }_{x} , {\theta }_{y}$: Rotor angular perturbations about the $x$ and $y$ axes respectively
$r$: The radial distance of a blade element from the rotor centre
$${r}_{tow}$$: Tower radius
$${\rho }_{air}$$: Air density
${\rho }_{tow}$: Tower material density
$R$: The radial location of the resultant thrust force on a blade
$T_{rotor}$: Rotor’s kinetic energy
$\hat{u}$: Non-inertial unit vector triad
$\hat{U}$: Inertial unit vector triad
$\nu_{tow}$: Disturbance in wind speed due to tower shadow
$$V_{tow}$$: Tower volume
$V_{0}$: Spatial mean wind speed
$V_{H}$: Hub height wind speed
$$V_{wind}$$: Wind speed
$V_{rel}$: Wind speed relative to the blade
$$r_{tow}$$: Tower radius
$$x_{tow}$$: Tower vibration
$$x_{t}$$: Tail vibration
$x_{R} , y_{R}$: Rotor’s translational degree of freedom along the $x$ and $y$ axes
$\tilde{X}$: Root mean square of $X$
$$\overline{X}$$: $$X/L_{r}^{2}$$
$$X^{*}$$: $$X/L_{r}$$

## Code availability

The codes used and/or analysed during the current study are available under^34^.

## References

[1] MacKay, D. J. C. *Sustainable Energy-without the Hot Air* (Bloomsbury Publishing, 2016).

[2] Mitchell, J. F. B. The ‘greenhouse’ effect and climate change. *Rev. Geophys.* **27**(1), 115–139 (1989).

[3] Paepe, R. et al. (eds) *Greenhouse Effect, Sea Level and Drought* Vol. 325 (Springer Science & Business Media, 2012).

[4] Burton, T., Sharpe, D., Jenkins, N. & Bossanyi, E. *Wind Energy Handbook* (John Wiley & Sons, 2011).

[5] IEC 61400-2. Wind turbines—Part 2: Design requirements for small wind turbines (International Electrotechnical Commission, 2013).

[6] Mathew, S. & Philip, G. S. (eds) *Advances in Wind Energy and Conversion Technology* Vol. 20 (Springer, 2011).

[7] Calautit, K. & Johnstone, C. State-of-the-art review of micro to small-scale wind energy harvesting technologies for building integration. *Energy Convers. Manag. X* **20**, 100457 (2023).

[8] Manwell, J. F., McGowan, J. G. & Rogers, A. L. *Wind Energy Explained: Theory* (John Wiley & Sons, 2010).

[9] Wilson, S. V. R., Clausen, P. D. & Wood, D. H. Gyroscopic moments on small wind turbine blades at high yaw rates. *Aust. J. Mech. Eng.* **5**(1), 1–8 (2008).

[10] Wright, A. K. & Wood, D. H. Yaw rate, rotor speed and gyroscopic loads on a small horizontal axis wind turbine. *Wind Eng.* **31**(3), 197–209 (2007).

[11] Hamdi, H., Mrad, C. & Nasri, R. Effects of gyroscopic coupling on the dynamics of a wind turbine blade with horizontal axis. In *Condition Monitoring of Machinery in Non-Stationary Operations: Proceedings of the Second International Conference, CMMNO’2012* (Springer, 2012).

[12] Hamdi, H. & Farah, K. Beam finite element model of a vibrate wind blade in large elastic deformation. *Wind Struct.* **26**(1), 25–34 (2018).

[13] Park, J.-H. et al. Linear vibration analysis of rotating wind-turbine blade. *Current Appl. Phys.* **10**(2), S332–S334 (2010).

[14] Hamdi, H. et al. Dynamic response of a horizontal axis wind turbine blade under aerodynamic, gravity and gyroscopic effects. *Appl. Acoust.* **86**, 154–164 (2014).

[15] Hamdi, H. et al. Static and dynamic study of a wind turbine blade with horizontal axis. *J. Environ. Sci. Eng.* **5**(9) 2011.

[16] Xiong, L. et al. Dynamic response analysis of the rotating blade of horizontal axis wind turbine. *Wind Eng.* **34**(5), 543–559 (2010).

[17] Da Costa, M. S. P. & Clausen, P. D. Structural analysis of a small wind turbine blade subjected to gyroscopic load. In *Journal of Physics: Conference Series* Vol. 1618 (IOP Publishing, 2020).

[18] Ou, Y. et al. Vibration-based monitoring of a small-scale wind turbine blade under varying climate conditions. Part I: An experimental benchmark. *Struct. Control Health Monit.* **28**(6), e2660 (2021). 35865081 10.1002/stc.2660 PMC9285914

[19] Tatsis, K. et al. Vibration-based monitoring of a small-scale wind turbine blade under varying climate and operational conditions. Part II: A numerical benchmark. *Struct. Control Health Monit.* **28**(6), e2734 (2021). 10.1002/stc.2660 PMC9285914 35865081

[20] Stol, K., Balas, M. & Bir, G. Floquet modal analysis of a teetered-rotor wind turbine. *J. Sol. Energy Eng.* **124**(4), 364–371 (2002).

[21] Velazquez, A. & Swartz, R. A. Gyroscopic effects of horizontal axis wind turbines using stochastic aeroelasticity via spinning finite elements. In *Smart Materials, Adaptive Structures and Intelligent Systems* Vol. 45097 (American Society of Mechanical Engineers, 2012).

[22] Castellani, F. et al. Experimental and numerical vibrational analysis of a horizontal-axis micro-wind turbine. *Energies* **11**(2), 456 (2018).

[23] Castellani, F. et al. The yawing behavior of horizontal-axis wind turbines: A numerical and experimental analysis. *Machines* **7**(1), 15 (2019).

[24] Vedovelli, M. et al. Experimental and numerical investigation of the effect of blades number on the dynamic response of a small horizontal-axis wind turbine. *Energies* **15**(23), 9134 (2022).

[25] Natili, F. et al. Experimental and signal processing techniques for fault diagnosis on a small horizontal-axis wind turbine generator. *Vibration* **2**(2), 187–200 (2019).

[26] Cai, H., Sun, Q. & Wood, D. Condition monitoring and fault diagnosis of a small permanent magnet generator. *Wind Eng.* **40**(3), 270–282 (2016).

[27] Velásquez, R. M. A. Bearings faults and limits in wind turbine generators. *Res. Eng.* **21**, 101891 (2024).

[28] Rao, S. S. *Mechanical Vibrations* (Prentice Hall, 2011).

[29] Sørensen, P., Hansen, A. D. & Rosas, P. A. C. Wind models for simulation of power fluctuations from wind farms. *J. Wind Eng. Ind. Aerodyn.* **90**(12–15), 1381–1402 (2002).

[30] Dolan, D. S. L. & Lehn, P. W. Simulation model of wind turbine 3p torque oscillations due to wind shear and tower shadow. In *2006 IEEE PES Power Systems Conference and Exposition* 1–6 (2006).

[31] Adams, M. L. *Rotating Machinery Vibration: From Analysis to Troubleshooting* (CRC Press, 2009).

[32] Muszynska, A. *Rotordynamics* (CRC Press, 2005).

[33] Schepers, J. G. et al. Lessons learned from 10 years of wind tunnel tests on small wind turbines designed by students. In *Journal of Physics: Conference Series* Vol. 2767 (IOP Publishing, 2024).

[34] Ramzy, A. Reduced order analytical modelling of micro wind turbine rotordynamics with tower shadow effects. Zenodo. 10.5281/zenodo.15313607 (2025).10.1038/s41598-025-00364-040360561

